# Multi-stakeholder use of patient experience data (PED): current state and future opportunities for the application of PED to inform decision-making

**DOI:** 10.1186/s41687-026-01030-3

**Published:** 2026-03-24

**Authors:** Chisom Kanu, Jessica L. Abel, Sarah L. Knight, Nicola Williamson, Calvin N. Ho, Ana Maria Rodriguez-Leboeuf, Chloe Carmichael, Katarzyna Wac, Rainer Herzog, Susan Vallow, Dorothee Oberdhan, Takako Kaneyasu, Olivier Chassany

**Affiliations:** 1 Eli Lilly & Company, Indianapolis, IN USA; 2 AbbVie, Inc., Florham Park, NJ USA; 3 Clarivate, London, UK; 4 UCB Pharma, Slough, UK; 5 Late Respiratory and Immunology, AstraZeneca, Gaithersburg, MD USA; 6 Global Preference and RW Practice Lead, Patient Centered Solutions, IQVIA, Montreal, Canada; 7 Quality of Life Technologies Lab, University of Geneva, Geneva, Switzerland; 8 School of Economics & Management, University of Geneva, Geneva, Switzerland; 9 Novartis Services Inc, East Hanover, NJ USA; 10 Faculty of Health Sciences and Wellbeing, University of Sunderland, Sunderland, UK; 11 Otsuka Pharmaceutical Development & Commercialization, Inc, Rockville, MD USA; 12 Department of Biomedical Sciences, College of Life Sciences, Ritsumeikan University Shiga, Shiga Prefecture, Japan; 13 Patient-Reported Outcomes Research (PROQOL), Health Economics Clinical Trial Unit (URC-ECO), Hotel-Dieu Hospital, AP-HP, Paris, France

**Keywords:** Patient experience data, Drug regulation, Health technology assessment, Patient engagement

## Abstract

Patient experience data (PED) encompass information on patient’s experiences, disease impact, and treatment preferences. PED are critical inputs to the drug development process and help ensure medicines are developed with the interests of patients in mind. The authors (all members of the Industry Special Interest Group and Regulatory and Health Technology Assessment Engagement Special Interest Group of the International Society for Quality of Life Research) have conducted a scoping review of how regulatory agencies, health technology assessment (HTA) bodies, private payers, clinicians, and patients currently use PED for decision-making. Recommendations are provided to address key challenges influencing multi-stakeholder use of PED.

## Introduction

Patient involvement in the drug development process began to achieve recognition in the 1980s when HIV/AIDs activists prompted the U.S. Food and Drug Administration (FDA) to establish a pathway for accelerated anti-retroviral therapy approval and outline a framework to capture feedback from HIV/AIDS advocates on clinical trial design, implementation, and evaluation [1]. Since then, there has been increasing emphasis globally on patient-centricity in medical product development and measuring what matters to patients. This has spurred the development of guidance documents by various regulatory agencies summarizing patient experience research principles to ensure patient-centric endpoints are meaningfully incorporated into drug development and evaluation [2–7] (US FDA 2009). Several regulators have also created systematic pathways and processes for sponsors to integrate patient experience data (PED) into drug development (e.g. FDA’s patient-focused drug development guidance series) and for patients to have their voices heard (e.g. the European Medicines Agency’s Patient and Consumer Working Party).

PED is broadly defined as any information that captures patients’ experiences and perspectives about their disease/condition and its treatment, its impact on their daily lives and quality of life, insights on outcomes important to them, and the relative significance of any issues as identified by patients [8]. This information can be collected directly from patients, clinicians, family members, and caregivers, and includes both qualitative data (via patient interviews, focus groups, ethnography, etc.) as well as quantitative data (via clinical outcome assessments [COAs], patient preference assessments, surveys, etc.). PED are increasingly being incorporated into medical product development to inform trial design, endpoint selection, regulatory benefit-risk assessments, and reimbursement decision-making. PED generated from clinical trials or real-world studies can provide valuable information on disease burden and treatment efficacy to support regulatory and reimbursement decisions, while also informing treatment decision-making for healthcare providers and patients post-approval.

Although the inclusion of PED in the regulatory process has spurred other stakeholders in the development lifecycle to consider the patient’s experience, how stakeholders use PED is generally not well-documented and constantly evolving. In this position piece, we outline the current state of PED usage among regulators, health technology assessment (HTA) bodies, payers, healthcare providers, and patients and highlight challenges and future opportunities for maximizing the use of PED to meet the needs of these stakeholders.

## Methods

This position article was informed by a structured scoping review and expert consensus. The authors systematically reviewed regulatory and HTA guidance documents, publicly available literature, and relevant reports to identify current practices and challenges in the use of PED by regulators, HTA bodies, payers, clinicians, and patients. Key themes and trends were extracted and synthesized through an iterative consensus process. Recommendations were developed through iterative discussion among the multi-stakeholder author group, reflecting collective expertise and current best practices.

## Current use of PED by key stakeholders

### Regulatory agencies

**Fig. 1:**
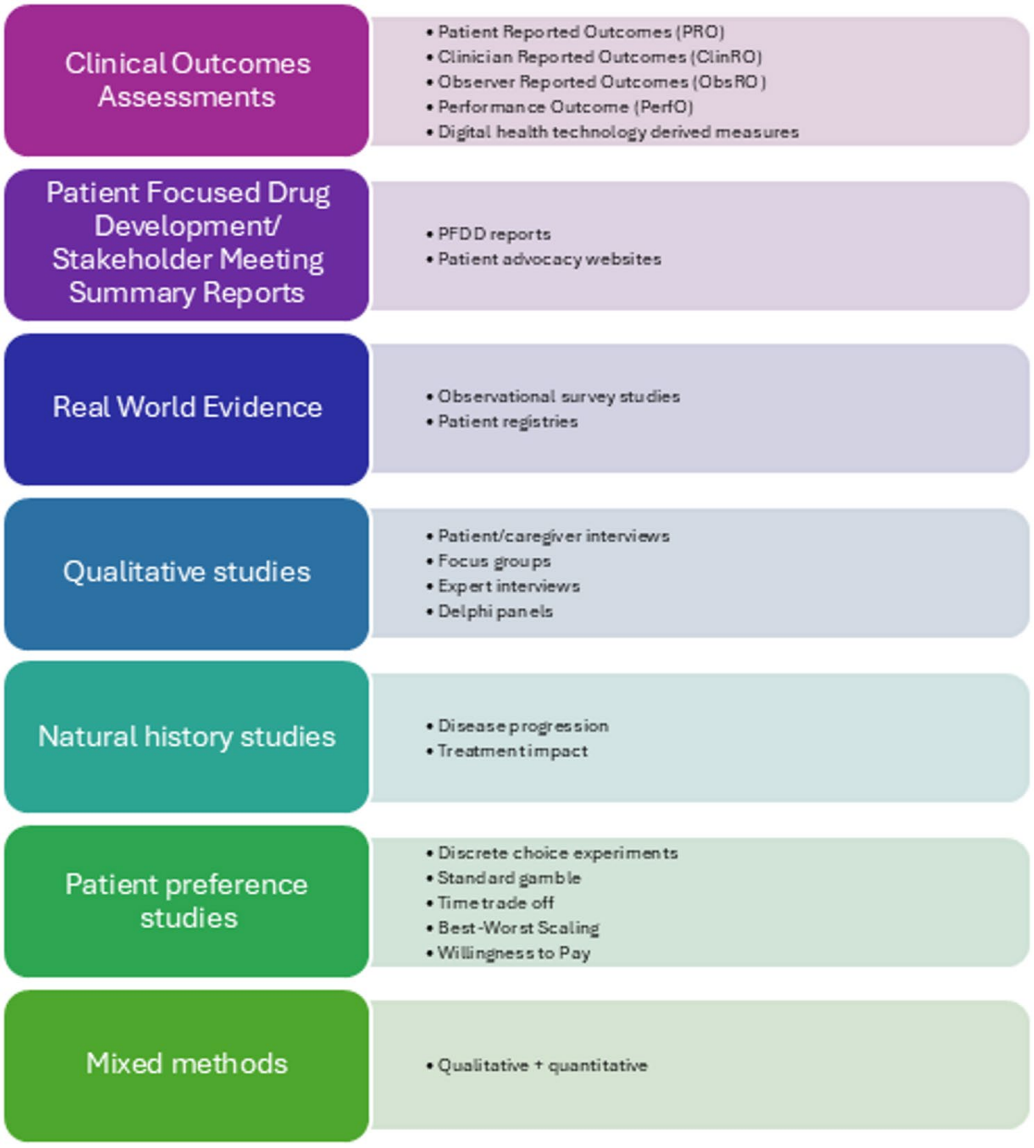
Summary of PED included in FDA applications—current use. *Other regulatory agencies use a subset of these PED

Global regulatory agencies use PED to inform the drug approval process and require sponsors intending to include PED in drug labeling to meet certain evidentiary standards. The US FDA has provided several guidance documents to facilitate scientifically rigorous collection of PED [9–12]; (2020), and since 2017 summarizes how PED was used for all approved applications in a table within their review documents. Furthermore, the Agency expects Sponsors to clearly communicate what PEDs were included in a submission in a similar PED table included as part of the Reviewer’s Guide of the application [13]. Figure 1 summarizes the types of PED typically included in regulatory applications to the FDA. A review of FDA approval documents from 2019 to 2023 noted most submitted PED was generated from COAs, with other types of PED (e.g., real-world, natural history and survey studies) appearing to be underutilized or under-reported [14]. A subsequent review of COA inclusion in FDA labels from 2012 to 2022 found COA use approximately doubled over the 10-year timeframe and, most commonly, COAs assessing signs and symptoms appeared in FDA labels [15]. While these reviews highlighted an emphasis on PED generated from COAs, the FDA has considered other types of PED in decision-making, as seen in the 2021 summary basis of approval (SBA) for odevixibat for pruritus in progressive familial intrahepatic cholestasis and the 2019 SBA for esketamine for treatment-resistant depression. In its assessment of odevixibat, the FDA used qualitative studies, observer-reported outcome (ObsRO) diary data, and insights from FDA patient listening sessions [16]. For esketamine, the FDA considered data from a patient preference study and COAs, as well as input from patient-focused drug development meetings in their benefit-risk assessment [17]. The FDA has also published guidance documents for incorporating Digital Health Technologies and Real-World Evidence in clinical drug development [18, 19].

The European Medicines Agency (EMA), which regulates medicine approvals for the European Economic Area, encourages developers to include PED in marketing authorization applications and offers scientific advice to facilitate successful generation, collection, and use of PED in medicines development and regulatory decision-making [2, 20]. Use of PED is highlighted in the example of, ritlecitinib wherein the Committee for Medicinal Products for Human Use (CHMP) of the EMA utilized a patient preference study and quantitative benefit-risk analysis in their evaluation to inform a positive opinion of ritlecitinib for the treatment of severe alopecia areata [21]. EMA has expressed its commitment to reinforcing patient relevance in evidence generation as a key priority in their 2025 Regulatory Science Strategy and recently issued a draft reflection paper outlining the European approach to PED [22, 23]. They have also recommended the use of PED in Post-Authorisation Safety Studies [24, 25].

The Medicines and Healthcare products Regulatory Agency (MHRA) in the United Kingdom has also produced guidelines on the use of PED in clinical trials and digital health product evaluations [26, 27]. The MHRA’s patient involvement strategy explicitly mentions PROs as an area of development for the agency and indicates plans to request COA data be submitted for licensing review [28]. Similarly, Swissmedic, which regulates medicine approvals in Switzerland, has a work plan to expand collaboration with patient and consumer organizations with the goal to incorporate patient experiences into Swissmedic’s processes [5]. Furthermore, a new regulation in Germany on the use of patient data facilitates the use of PED by German regulators [29, 30].

The Pharmaceuticals and Medical Devices Agency (PMDA) in Japan has published a guidance document which includes a framework for capturing PED and highlights the value PMDA places on understanding the patient experience [6]. In China, the Center for Drug Evaluation of the National Medical Products Administration released guidance on the use of PROs in clinical trials of new drugs [7], largely aligned with current methodological standards and best practices with an emphasis on ensuring cultural and linguistic validation of questionnaires used for patients in China. They have also launched a Patient-Centered Rare Disease Drug Development Pilot Program to guide applicants in the incorporation of patient perspectives in drug development for rare diseases to inform benefit-risk evaluation and foster collaboration between regulatory agencies, drug developers, patients, and other stakeholders [31].

Although the majority of regulators focus on PED generated from COAs used in clinical trials, there is increasing openness to considering PED derived from other sources, such as prospective observational studies [18, 32] and qualitative interviews [33]. Data generated from wearables and other sensor-based technology may also be considered as additional sources of PED [34]. Furthermore, patient preference studies can provide insights on important aspects of a treatment from the patient perspective [35–37], with their use supported by several guidance documents [36, 38–42], and their impact on decision-making increasing as seen in the examples of esketamine [17], and ritlecitinib [21]. These studies may provide added value where patients need to decide between different treatment options, and when a treatment is being indicated for a heterogenous population [33].

The use of qualitative in-trial interviews has also increased to better understand the patient experience of a disease following treatment in a trial [43, 44], to identify or confirm important concepts, evaluate changes in symptoms or functioning following treatment, and explore meaningful within-patient changes in outcomes qualitatively to support interpretation of COA scores [45]. This methodology is being requested by FDA more frequently to generate PED, especially with respect to understanding patient perspectives on meaningful change in patient-centric endpoints in clinical development programs [10]. These data have also been used by regulators and HTA bodies to inform decision-making [46–48].

Wearable technology, such as heart rate sensors and accelerometers, has advanced PED collection as it relates to bio-physiology and physical functioning, and enables continuous, real-time data collection and analysis of objective data [34]. These devices provide detailed insights into individual behaviors and their impact on health and life quality [49], often complementing PED generated from traditional COAs.

While there is an emphasis across regulators on the importance of PED, no guidance exists for the use of PED generated from prospective real-world studies.

### Health technology assessment (HTA) bodies

Little guidance exists on whether and how HTA bodies use PED to inform their decision-making. Overall, expectations on PED to support reimbursement typically focus on health-related quality of life (HRQoL)/PRO data, with some guidance available on the role of PROs in HTA [50] but limited interest across agencies in PED beyond that generated from PROs in clinical trials and real-world settings [51]. Typically, PRO data are evaluated on a case-by-case basis, with quality of evidence being of paramount importance. PED is increasingly used by HTAs to support clinical efficacy, inform on burden of illness, qualify and quantify the added value of new treatments from the patients’ perspective, and support health economic models [33]. A recent study analyzing HTA appraisal reports containing PRO endpoints from Canadian, French, German, Scottish, and British HTA authorities found considerable variability in HTA evaluation of PROs, with the majority of PRO data submitted receiving unfavorable assessments, primarily due to the absence of a predefined responder analysis, use of non-validated PROs, and uncertainty concerning the meaningfulness of changes in PRO scores [52]. Additionally, a review of COA inclusion in HTAs of German, French and English bodies from 2012 to 2022 found an increase from 45.4% to 64.9% of non-mandatory COA inclusions, defined as use beyond primary endpoints or COAs essential for cost-effectiveness, which mostly assessed health status or quality of life [15].

In cost-effectiveness markets that use economic evaluations in decision-making (e.g., UK, Canada, Australia, Netherlands, Sweden, Belgium, Norway, Denmark, Japan), health state utilities derived from generic preference-based measures, such as EQ-5D or disease-specific PRO measures, may be used as inputs to health economic models for cost-effectiveness assessments to capture patient and caregiver perspectives [53–57]; (2015a). In comparative clinical effectiveness markets like Germany, PRO data may be considered as evidence for clinical value. Specific guidance on PROs from clinical trials is included as part of IQWiG’s General Methods, and PROs are considered by IQWIG and G-BA under morbidity and HRQoL categories for benefit assessments, with HRQoL data required by German law for decision-making in line with the 2013 European Network for Health Technology Assessment (EUnetHTA) guidelines [58–61].

However, the acceptance of PRO data by IQWIG and G-BA is challenged by the inclusion of PRO-based endpoints as exploratory without pre-specified analyses, large amounts of missing data [62], unvalidated PROs, difficulties interpreting change across treatment groups, and lack of long-term follow up post-progression. PRO data from double-blind studies are preferred, but data from open-label studies may inform benefit ratings when blinding is not possible [62]. IQWIG has also implemented its own guidelines on thresholds for meaningful change, considering a clinically meaningful within-patient change threshold for responder definitions to be ≥ 15% of the scale range [58]. PED are also required for the dynamic HTA of digital health applications (DiGA) in Germany, which must demonstrate safety, functional capability, and quality requirements, as well as positive care effects defined in the DiGA regulation or Digital care applications regulation to obtain approval for reimbursement [63–65].

In France, HRQoL data are considered important by the French National Authority for Health (Haute Autorité de Santé; HAS) but must meet strict criteria for the evidence to be considered as a driver for added clinical value [66]. As outlined in the HAS Transparency Committee doctrine, HRQoL data can contribute to the assessment of a product’s clinical effect as in the example of ivacaftor/tezacaftor/elexacaftor for cystic fibrosis [67]. Positive efficacy and safety data along with an improvement in HRQoL could lead to a clinical added value (CAV) level higher than V (no improvement) if validated COAs are used following a robust methodology (i.e., pre-specified endpoints, hierarchical testing, clinically meaningful within-patient change pre-specified, double-blind conditions, and appropriate analyses with minimal missing data). Importantly, lack of HRQoL data may result in a negative impact on the CAV if HAS expects to see HRQoL data, particularly for chronic, debilitating and terminal diseases [68, 69].

In the UK, the National Institute for Health and Care Excellence (NICE) technology evaluations manual [70] outlines their methods and processes for health technology evaluations. While economic evaluations are a key focus, the manual also highlights the importance of qualitative research in submissions to describe patients’ experiences and quality of life with a disease/condition and its treatment, and expert elicitation or expert opinion. The agency has quality appraisal checklists to assess economic evaluations, quantitative studies (reporting correlations and associations) and qualitative studies as part of its public health guidance [71]. Additionally, NICE provides an evidence framework for DHTs and recommends COAs in comparative studies to support their evidence of effectiveness [72]. 

### Private Payers in the United States

A lack of clarity exists on current expectations and evidentiary requirements for PED to inform payer decision-making in the US. Based on a 2020 survey of US payer perceptions of the use of PRO evidence in oncology to inform formulary decision-making, payers view PRO evidence (which is the most commonly available PED) as useful for supplementing other clinical trial data [73]. However, given the lack of familiarity with PED among payers, significant opportunities exist to educate these stakeholders on the measurement science of COAs to generate PED and the overall value of PED in medical product development to drive recognition and use of these data as a key component in payer decision-making.

### Patients, patient advocacy groups, and clinicians

While there is increasing recognition and use of PED by regulators, HTA bodies, and payers, limited PED generated during drug development has traditionally been communicated back to patients, advocacy groups, or clinicians to support their treatment decision-making. In markets where direct-to-consumer advertisements are allowed, PED often reaches patients through messages focusing on the benefits of specific treatments without providing an understanding of conditions or treatment outcomes [74–76]. For clinicians, PED are typically communicated in the form of scientific publications, conference disclosures, and drug labels, which typically does not provide the full context or detail of the patient experience. As demonstrated by Doyle et al., communication of information, good provider–patient relationships, and patients’ agreement with the need for treatment are determinants of treatment effectiveness and adherence [77]. While the PROTEUS consortium provides specific recommendations for the dissemination of PRO evidence collected back to patients [78], there remains a need for appropriate channels to communicate accurate and non-misleading PED to patients and patient advocacy groups to inform their overall understanding of their disease/condition and support treatment decision-making.

## Current challenges and future opportunities for the application of PED

Despite advances in the use of PED by various stakeholders, challenges for further integration and acceptance of PED remain. Greater transparency, harmonization, and consistency are needed across and within regulators, HTA bodies, and payers on evidentiary requirements and expectations for inclusion of PED in submissions to facilitate decision-making. For instance, while the proportion of new drug applications to FDA that include PED has increased between 2020 and 2024, there is very little publicly available data on how the agency considers that PED during reviews [79]. In the HTA arena, there is an opportunity to address methodological issues that assessment agencies have identified as barriers to considering PED more seriously [80]. Without additional transparency and harmonization, it is challenging for sponsors to develop comprehensive strategies to meet different stakeholder requirements within a development program and may lead to increased burden for trial participants in completing multiple COAs assessing overlapping concepts and in the collection of PED that does not adequately reflect outcomes that matter to patients [62, 81].

Several initiatives by regulators and HTA bodies aim to enhance the use of PED to inform decision-making (Table 1). The US FDA’s Patient-Focused Drug Development guidance series was developed to support generation of high-quality PED for a regulatory context, and the Agency continues to emphasize the importance of quality PED to inform benefit-risk decision-making. Additionally, the Centers for Medicare & Medicaid Services (CMS) has recognized the importance of the patient perspective in drug price negotiations under the Inflation Reduction Act and has established key channels for patients and caregivers to participate in the process, inclusive of submitting written data to CMS, participating in patient round tables, and engaging in clinician town halls [84, 85].

**Table 1:** Summary of available guidance and key considerations for PED evidence generation across regulatory agencies and HTA bodies

Stakeholder (country)	Guidance	Key Considerations
**Regulatory Agencies**		
FDA (United States)*	• US FDA Patient-Focused Drug Development Guidance Series [9–11, 13] • Benefit-Risk Assessment for New Drug and Biological Products [82] • Framework for FDA’s Real-World Evidence Program [18] • Framework for the use of digital health technologies in drug and biological product development (FDA, 2023c)	• 4-part methodological guidance series outlines stepwise approach to how stakeholders can systematically collect, submit, and meaningfully incorporate PED to inform product development and regulatory decision-making [9–11, 13] • PED are an essential component to assessment of the benefit-risk framework and FDA is required to report on the use of PED in regulatory decision making [82] • Validated COAs assessing signs, symptoms or functioning, pre-specified primary or secondary endpoints, multiplicity adjusted, meaningful within-patient change thresholds, double blind design preferred or open-label with external comparator arm [11, 82] • Other PED may justify COA endpoint, support interpretation of endpoint, and inform benefit-risk [10, 82] • Guidance documents for incorporating DHTs and RWE in clinical drug development (FDA, 2018, 2023c)
EMA (EU plus Iceland, Norway, and Liechtenstein)	• Reflection paper on the regulatory guidance for the use of health-related quality of life (HRQL) measures in the evaluation of medicinal products [2], • The use of PRO measures in oncology studies [20], • Patient experience data in EU medicines development regulatory decision-making (EMA [4]), • Guideline on good pharmacovigilance practices (GVP): Module VIII – Post-authorisation safety studies [24], • EMA policy on access to EudraVigilance data for medicinal products for human use [25],	• Current guidance provides broad recommendations for the use of HRQoL in drug development and evaluation [2, 20] • COAs assessing signs, symptoms, functioning, or HRQoL, pre-specified endpoints, validation of COAs ahead of pivotal studies preferred. Other types of PED may be considered in benefit-risk assessments [2, 20]; (EMA 2022) • Draft reflection paper highlights EU regulatory approach to PED [23] • Recommendations on the use of PED in post-authorization safety studies [24, 25],
MHRA (United Kingdom)	• MHRA guidance on the use of real-world data in clinical studies to support regulatory decisions [28] • Guidance: Patient-reported outcomes and experiences study [26]	• Guidance on the use of PED in clinical trials and digital health product evaluations [26, 28]
PMDA (Japan)	• Guidance on patient participation [6],	• It is necessary to implement COAs in clinical trials based on scientific methods indicated in guidelines and to consider clinical significance of the results [6]
• Center for Drug Evaluation of the National Medical Products Administration (China)	• Guidelines for the usage of patient-reported outcomes in clinical research for implementation on a trial basis [7],	• Provides specific guidance on the validation of PROs for use in clinical research, largely congruent with earlier FDA guidances [7]
**HTA Bodies**		
EUnetHTA (EU HTA bodies)	• Endpoints used for relative effectiveness assessment: health-related quality of life and utility measures [56]; clinical endpoints [57] Guidance on patient and healthcare professional involvement [83]	• Methodological considerations and challenges encountered by HTA bodies when performing rapid relative effectiveness assessments. Recommendations on the use of clinical endpoints and HRQoL and utility measures in clinical trials [56, 57] Recommendations for involving patient and healthcare professionals in HTA assessments [83]
HTA CG (EU HTA bodies)	• Guidance on outcomes for joint clinical assessments (HTA CG 2024)	• Guidance for member states in defining relevant outcomes for the scoping process, and to help assessors to assess and report all elements that member states need for the national appraisal of the clinical added value of a health technology (HTA CG, 2024)
HAS (France)	• Transparency Committee doctrine: Principles of medicinal product assessments and appraisal for reimbursement purpose [68]	COA data are important but must meet specific requirements: (1) pre-specified endpoints, included in hierarchical testing, (2) validated COAs, (3) pre-defined clinically meaningful change thresholds, (4) appropriate statistical analyses, (5) minimal missing data, (6) double-blind trial design [68] • Improvement in HRQoL measure could lead to higher clinical added value [68]
IQWiG/G-BA (Germany)	• IQWiG General Methods Version 7.0 of 19 September 2023 [58], • Gemeinsamer Bundesausschuss. G-BA rules of procedure [60], • Gemeinsamer Bundesausschuss. G-BA submission dossier module [61, 64].	• COA data considered under morbidity and HRQoL categories for benefit assessments, HRQoL data required by German law for decision-making [58, 60, 61], • Requirements to have pre-specified primary or secondary endpoint, pre-specified analyses, minimal missing data, validated COAs [58] • Double blind trial design preferred, open label design may be acceptable if blinding is not possible [58], • Clinically meaningful within-patient change threshold for responder definitions to be ≥ 15% of the scale range to achieve additional benefit rating for an intervention [58], • Pathway for digital health technologies to be prescribed and reimbursed [64]
NICE (United Kingdom)	• NICE health technology evaluations: the manual [70] • Methods for the development of NICE public health guidance [71] • Evidence standards framework for digital health technologies [72]	• EQ-5D is preferred to generate health state utilities for cost-effectiveness assessments. Other methods acceptable if EQ-5D is not appropriate [70], • Methods and processes for health technology evaluations detailed in the manual, including quality appraisal checklists for economic evaluations and quantitative and qualitative studies [71] • Evidence framework for DHTs recommends including COAs in comparative studies to support evidence of effectiveness of DHTs [72],
Central Social Insurance Medical Council (Japan)	• Guideline for cost-effectiveness evaluation in Japan [54]	• Recommended to use health state utilities derived from the Japanese EQ-5D-5 L to reflect preferences of the general Japanese population [54] • Recommendation to prioritize patient responses and limit proxy responses by family members or caregivers [54] • When alternative methods are used, it is necessary to demonstrate that the results obtained through those methods align with patient preferences [54]

C_2_H, Center for Outcomes Research and Economic Evaluation for Health; DHT, digital health technology; EMA, European Medicines Association; EUnetHTA, European Network for Health Technology Assessment; FDA, Food and Drug Administration; G-BA, German Federal Joint Committee/Gemeinsamer Bundesausschuss; HAS, Haute Autorité de santé; HTA CG, member state coordination group on health technology assessment; IQWIG, Institute for Quality and Efficiency in Health Care; JCA, Joint Clinical Assessment; MHRA, Medicines and Healthcare products Regulatory Agency; NICE, National Institute for Health and Care Excellence; PMDA, Pharmaceuticals and Medical Devices Agency; RWE, Real World Evidence

*Other guidance documents are in existence, but these are the key/most recent documents

In the European setting, the EMA’s CHMP assessment report template has been updated to indicate what PED is assessed by regulators, providing increasing transparency and a structured overview of different types of PED that have been submitted and used in evaluations [23]. Furthermore, the implementation of the Joint Clinical Assessment aims to create a streamlined process across HTA bodies on comparative effectiveness and safety of investigational products, with an opportunity to include PED in the submission dossier to demonstrate unmet need and burden of disease and justify concepts for measurement to inform reimbursement decision-making [29, 83]. There is guidance on outcomes for joint clinical assessments that have been adapted based on methodological guidelines created under EUnetHTA activities (EUnetHTA [56, 57], Member State Coordination Group on Health Technology Assessment [86]). Importantly, however, decisions regarding pricing and reimbursement will remain with individual HTA bodies [33].

Among sponsors, the generation of PED is often siloed within functional areas and duplicative across teams, underscoring the need for a more integrated, cross-functional approach. Such coordination could enhance operational efficiency, maximize the utility of collected data, reduce patient burden, and more effectively address the needs of stakeholders [35]. Given different types of PED can be complementary and similar evidence generation methods may be appropriate to address a range of research questions, integrated evidence generation and communication strategies must thoughtfully consider which PED best align with key stakeholder requirements across the product development lifecycle to generate robust data for decision-making. For example, insights from a literature review and qualitative concept elicitation interviews to identify important signs/symptoms and impacts on daily life can not only inform selection of key outcomes but also help identify important potential treatment attributes to be explored in future research [87].

While this article has focused on the use of PED, it cannot be viewed in isolation and many stakeholders harness patient engagement to obtain valuable insights from patients, which we direct the reader to here [88, 89, 22, 90–95]; (EUnetHTA 2023). A recent landscape review of patient engagement and PED initiatives in regulatory and HTA [96] highlighted various opportunities underway to involve patients. They concluded that guidelines have often been put in place in isolation, and policies combining patient engagement and PED generation may be beneficial. They note few working examples exist where guidelines have been integrated, and PED alone, without patient engagement, is likely to be of lower quality. An important initiative to overcome challenges with disintegration of patient engagement and PED, is the Global Patient Experience Data Navigator, co-created by the Patient Focused Medicines Development. The publicly available toolkit was developed using a multi-stakeholder and disease-agnostic approach to provide resources to maximize the efficient patient engagement and collection of PED for patient benefit and use by multiple stakeholders (Wilgoss et al. 2023). It provides an overview of approaches to identify what outcomes matter most to patients and families, what tools are appropriate outcome measures, and how PED is used by stakeholders throughout product development and the healthcare process (Wilgoss et al. 2023).

### Summary and recommendations

The increasing focus on patient-centric research and associated methods in the drug development process has largely been driven by recent regulatory guidances describing the use of COA-based endpoints in clinical trials. While stakeholders other than regulators, such as HTA bodies and payers, have also considered PED in their decision-making, expectations and evidentiary requirements for the use of PED vary. Greater clarity from regulators and HTA bodies in their expectations and requirements for PED and harmonization across key stakeholders is not only welcome but necessary for the development of efficient PED evidence generation strategies across the drug development cycle.

It is important to be intentional and systematic at the outset in how PED is collected and used across product development to ensure data are of value and can be used as scientific evidence to inform decision-making across a broad range of stakeholders. Harnessing generation of PED for multiple purposes and stakeholders can not only ensure more efficient PED evidence generation but also help sponsors prioritise research activities whilst engaging with various stakeholders early on, as recommended by both regulators and HTA bodies. To support a purposeful, planned, and integrated PED evidence strategy, key stakeholders (like regulatory agencies and HTA bodies) need to be clearer on the types and volume of data and preferred methods for PED collection prioritised for decision-making to ensure selection of methods is informed by evidence needs and aligned with requirements of all key stakeholders. This can also help to prespecify the different uses of PED in study protocols and analysis plans [80]. A more coordinated PED strategy across the lifecycle and for various stakeholders would not only facilitate PED evidence planning and reduce burden on trial participants but also enable sponsors to invest in determining how to maximise PED evidence use beyond a regulatory context by patients, patient communities, and clinicians to support patient-centered clinical practice.

In addition to the need to engage regulators and reimbursement authorities early and throughout the product lifecycle, engaging patients early and throughout clinical development programs as both participants and informed decision-makers in PED strategy planning will maximise the robustness of both the patient voice and patient engagement initiatives and ensure adoption of a patient-focused approach to drug development.

### Limitations

This article is not without limitations. While the intention was to provide an overview of multistakeholder use of PED across regulators, HTA bodies, payers, patients, and clinicians, it was not possible to comprehensively and systematically review all initiatives and use cases of PED. Therefore, the article focused on the largest regulators and HTA bodies, and it is possible that there are uses of PED by other regulators, HTA bodies, and other stakeholders that have not been mentioned here.

## Conclusions

Despite several initiatives and guidelines, variability exists within and between regulators and HTA bodies in what they are willing to accept regarding PED. There is a need for harmonization in expectations for PED and need for increased clarity from regulators, HTA bodies, and payers on how PED is integrated into their decision-making. Sponsors should aim to better align PED evidence generation and communication strategies to address key stakeholder needs across the product development lifecycle. Clear and consistent communication of PED to patients, patient advocacy groups, and clinicians is also needed to enhance understanding of patient experience with a disease/condition and better inform treatment decision-making.

## Data Availability

Not applicable.
